# Extending regular oral hygiene reduces the incidence of upper respiratory symptoms

**DOI:** 10.1590/1807-3107bor-2025.vol39.118

**Published:** 2025-11-07

**Authors:** Cassiano Kuchenbecker RÖSING, Juliano CAVAGNI, Isadora dos Santos ROTTA, Lorena Lirio SOSSAI, Rodrigo de Oliveira CAETANO, Gabrielle PEDRONI, Stephanie Anagnostopoulos FRIEDRICH, Bernal STEWART, Zilson MALHEIROS, Carlos BENÍTEZ, Lyndsay SCHAEFFER

**Affiliations:** (a) Universidade Federal do Rio Grande do Sul – UFRGS, School of Dentistry, Department of Periodontology, Federal University of Rio Grande do Sul, Porto Alegre, RS, Brazil.; (b) Colgate-Palmolive Company, Piscataway, NJ, USA.; (c) Latin American Oral Health Association, São Paulo, SP, Brazil.

**Keywords:** Oral Sprays, Mouthwashes, Influenza, Human, Respiratory Tract Infections

## Abstract

In this randomized controlled clinical study, the effectiveness of two different modes of extending oral hygiene - either gargling or using an oral spray - on the self-reported incidence of upper respiratory symptoms were evaluated, to help with the prevention of respiratory symptoms. The study was conducted in Porto Alegre, Brazil. A total of 205 individuals were enrolled and assigned to one of three groups: Control Group: brushed twice daily for two minutes with a commercially available fluoride toothpaste; Gargling Group: brushed twice daily for two minutes with a commercially available fluoride toothpaste and then gargled with a mouthwash containing 0.075% CPC (cetylpiridinium chloride); and Oral Spray Group: brushed twice daily for two minutes with a commercially available fluoride toothpaste and instructed to use an oral spray containing 0.075% CPC (3 pumps of spray applied directly into their mouth) twice daily. All subjects were asked to complete the WURSS-21 Daily Symptom Report via a diary for the 3 months duration of the study. Based on analysis of the diaries, the use of regular oral hygiene with brushing, in addition to a type that extended to the entire oral cavity, reduced the incidence and severity of upper respiratory symptoms throughout the duration of the study. Data were statistically analyzed using ANOVA (α = 0.05). Individuals using either mouthwash or spray reported approximately 20% fewer days with respiratory symptoms compared with the control group. Regular oral hygiene that extended to the whole oral cavity with either a mouthwash or an oral spray may help to reduce the incidence of upper respiratory symptoms.

## Introduction

Seasonal upper respiratory infections are responsible for high degrees of morbidity and mortality around the world. Among respiratory infections, those of viral origin, especially caused by the Influenza viruses are the most common. Seasonal flu accounts for an important impact on health systems.^1^

During the COVID-19 pandemic, interest in respiratory infections and their related symptoms increased dramatically and a number of studies were conducted to understand the role of different approaches that could mitigate the effects of respiratory infections.^2^The most common respiratory infections are both of viral and bacterial origin. In fact, frequently they are a mix of microbiological origin. In this sense, cleaning measures have the potential of impacting microbial load and, therefore, preventing these infections. Cetylpiridinium chloride has a broad antimicrobial spectrum and could, therefore, be of interest in this connection.

To control both influenza and COVID-19 outbreaks, vaccination has been demonstrated to be a preventive and risk mitigation strategy with satisfactory degrees of effectiveness, both individually and collectively.^3,4^ However, in addition to vaccination, different strategies have been proposed. Specifically in terms of the oral cavity, studies have been conducted with previously studied antimicrobial agents to assess their antiviral effectiveness. Chlorhexidine and cetypliridinium chloride have been tested for their antiviral capacity and have demonstrated efficacy both in vitro and in vivo. However, the body of evidence is not robust, warranting additional studies.^5,6^ In this sense, adjuncts to oral hygiene were assessed relative to their potential impact on viral infections, if any, since the oropharyngeal region is one of the possible portals of entry for infection.

In the same manner as hygiene of the hands is an effective measure in dissemination of a series of infections – including viral types, it was hypothesized that oral hygiene measures could also provide these same benefits.^7^ For this reason, oral antiseptics have been studied for their ability to neutralize viruses. For example, we evaluated the virucidal capacity of chlorhexidine in a systematic review and observed a virucidal effect on influenza when used in mouthwashes.^5^

Other studies have evaluated the potential of solutions used locally in the mouth/oropharyngeal region to combat viral infections. In an in vitro study, using two commercially available toothpastes and mouthwashes, it was demonstrated that products containing hydrogen peroxide, cetylpiridinium chloride (CPC) or stannous fluoride demonstrated antiviral effects. However, further clinical studies are warranted to ascertain whether this effect translates into a meaningful benefit to the consumer.^8,9^

CPC is a quaternary ammonium compound, with excellent safety and has been disseminated for use as an adjunct to oral hygiene. It has good levels of compliance relative to use by patients and its antimicrobial effect against bacteria in the oral microbiome is measurable.^10^ While it has been demonstrated effective as a pre-procedural rinse in dental office treatments,^11^ its antiviral effect needs additional studies.

Leveraging existing oral hygiene products for their antimicrobial effects, the present study compared two means of delivering oral cleaning that included the whole oral cavity. The general hypothesis was that using CPC would decrease the occurrence of upper respiratory symptoms. Moreover, it was hypothesized that the form of administration could generate differences in effect.

The aim of this randomized controlled clinical trial was to evaluate the effect of extending regular oral hygiene with either a mouthwash (gargling) or an oral spray CPC on the incidence of upper respiratory symptoms.

## Methods

### Study design

This study was a phase III, randomized, one-center, parallel group, open label, controlled clinical study. The study was conducted at the Federal University of Rio Grande do Sul, in Brazil. The present study has been reported according to the CONSORT guidelines.

### Ethical considerations

The present study was approved by the Institutional Review Board of the Federal University of Rio Grande do Sul (Protocol #5.425.383) and was conducted in accordance with the principles stated in the Declaration of Helsinki. All participants read and signed the Term of free and informed consent before the study began.

### Sample size estimate

The main outcome was the presence of upper respiratory symptoms. Sample size calculation was performed to detect a difference of 3% in the proportion of subjects exhibiting at least one respiratory symptom. A within group standard deviation of 0.53 was assumed from a pilot study, with 80% power. The pilot exercise was performed with individuals from the same age group and from the same area as that of the individuals included in the study. Then, it was estimated that 180 individuals (60 per group) would be necessary to detect a difference with alpha and beta errors of 0.8 and 0.05, respectively. Taking into consideration possible attrition, 75 subjects per group (225 total) were recruited to meet the goal of 60 individuals per group to complete the study.

### Inclusion/non-inclusion/exclusion criteria

Participants were invited to participate in the trial by means of folders distributed in universities and health units. Subjects who met the inclusion/non-inclusion criteria and signed the Term of Informed Consent entered the study. The following criteria were adopted: subjects aged 18–70 years, including both males and females, participated in the study. Inclusion criteria stipulated good general health (absence of any disease/condition that would influence the risk of having upper respiratory infections, such as immunodeficiency, presence of chronic pulmonary disease, etc.) and availability for a period of months. Subjects were not included if they presented any of the following: participating in another clinical study; pregnant or lactating women; allergic to oral care, personal hygiene products or their ingredients; users of anesthetic oral sprays or presenting irritative oral lesions, diabetes, presenting immune incompetency (HIV, AIDS, use of immuno-suppressive drugs); under extensive dental treatment, including oral surgery; full denture users; presenting carpal tunnel syndrome or arthritis in the hands.

During the study, individuals presenting the following characteristics/situations were excluded: subjects that failed to comply with the protocol requirements; subjects treated with medication(s) during the course of the study that could interfere with the parameters under study; subject that received emergency dental or medical treatment that may interfere with the parameters under study; subjects that presented adverse events probably related to the protocol; women that became pregnant during the course of the study.

### Experimental procedures

The present study was conducted in the city of Porto Alegre, south Brazil from July to the beginning of October 2022. Porto Alegre is located in the latitude parallel 30^o^South. This period is the Winter season and coincides with the highest occurrence of upper respiratory tract infections. The temperature during these months mostly ranges from 0 – 15^o^C.

Subjects were screened by the dental examiner to identify those who met the inclusion/non-inclusion criteria. A randomization list was computer generated in blocks of 15 with the aid of the site www.randomization.org by an external researcher. Clinical staff members were not aware of the random allocation list. Individuals were randomly assigned to one of the following groups:

a.Control group: Subjects were provided with an adult soft-bristled toothbrush and a tube of a conventional non-antibacterial toothpaste (1,450 ppm MFP). They were instructed to brush their teeth for two minutes twice daily (in the morning and evening) with the toothpaste provided.

b.Gargling group: Subjects in this regimen were provided with an adult soft-bristled toothbrush, mouthwash and a tube of conventional non-antibacterial toothpaste (1,450 ppm MFP). They were instructed to brush their teeth for two minutes twice daily (in the morning and evening) with the toothpaste provided. After brushing their teeth, subjects were instructed to gargle their mouth for 30 seconds, using 20 ml of the mouthwash provided (0.075% CPC). After gargling, they should spit out the solution.

c.Oral spray group: Subjects in this regimen were provided with an adult soft-bristled toothbrush, oral spray containing 0.075% CPC, and a tube of conventional non-antibacterial toothpaste (1,450 ppm MFP). They were instructed to brush their teeth for two minutes twice daily (in the morning and evening) with the toothpaste provided. After brushing their teeth, subjects were instructed to use the oral spray by applying 3 pumps of spray directly into their mouth.

Products were distributed in an area separately from the examination room by site personnel not involved in the clinical evaluations. Products were dispensed in a sealed opaque bag to account for any differences in product esthetics, and packaging among study groups. The study lasted for 90 days and was conducted from the beginning of July to beginning of October of 2022.

At baseline, subjects completed a health questionnaire on their medical and dental history. The subjects’ health questionnaires were updated at subsequent visits. Clinical evaluations at all stages included assessments of soft and hard tissues. Individuals presented to the research facility at baseline, and at intervals of 30 and 90 days for product distribution. Compliance with product use was checked by return of the non-used products.

### Main outcome

The main outcome of the present study was the WURSS-21 (Wisconsin Upper Respiratory Symptom Survey- 21).^12^ The WURSS-21 questionnaire was originally developed and validated for the North American Population.

The WURSS-21 questionnaire consists of 21 questions about upper respiratory symptoms, which if present, are also qualified in terms of severity (very mild, mild, moderate, severe). For this purpose, a 7-point Likert scale was used. Moreover, there are questions about the evolution of the situation in comparison with the previous day. There was no need for any adaptation of the translated questionnaire. Participants received individualized instructions on how to answer the questionnaire and, in need of anything, they could contact the research team.

At baseline, subjects were provided with a paper-based WURSS-21 instrument and instructed to complete it daily for a period of 90 days. Electronic copies of the completed questionnaires were submitted to the research team weekly, in the form of photographs. On conclusion of the study, participants returned the physical notebooks.

### Statistical analysis

Data analysis was performed with the use of SPSS. The raw symptom severity, intensity score data were rescaled to a binary presence/absence score for statistical analysis. Interval plots with 95% confidence intervals were created to facilitate between treatment group symptom rate comparisons. One-way analysis of variance (ANOVA) was used to make statistical comparisons between the symptom rates of the treatment groups. A Group p value less than 0.05 in the ANOVA indicated statistical significance. If a significant group effect was detected, a Tukey multiple comparison test was used to assess pairwise differences among the treatment groups.

## Results

**Figure 1:**
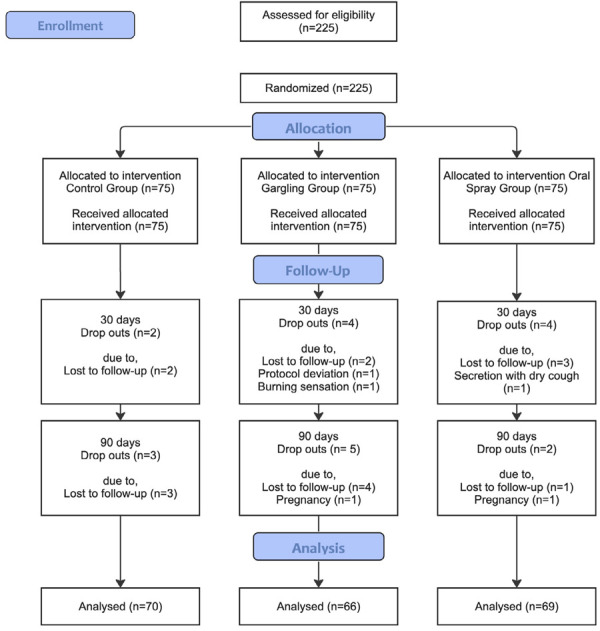
Consort flow diagram.

The present study included 225 individuals of whom 205 concluded the protocol (108 of men and 117 women) with an average age of 33.15 years, ranging from 18 to 69 years. Of these, 66 were in the Gargling Group (29 men; 37 women), 69 in the Oral Spray Group (35 men; 34 women) and 70 were in the Control Group (34 men, 36 women). Figure 1 presents the flow diagram of the study.

**Figure 2:**
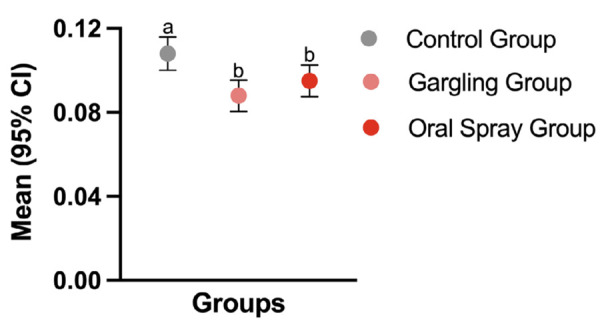
Mean (95%CI) of positive answers to the question: How sick do you feel today?

The main outcome of the present study is presented in Figure 2. It complies with the answer to the question: How sick do you feel today? If the individual presented any upper respiratory symptoms, he/she was considered positive relative to this question. Taking into consideration the daily symptoms of the participants over the course of the study period, it could be observed that both test groups (regular gargling or spraying) generated fewer positive answers for feeling sick (p < 0.05), when compared with the control group.

Different small letters indicate a statically significant difference between groups (p ≤ 0.001). CI: confidence interval.

**Figure 3:**
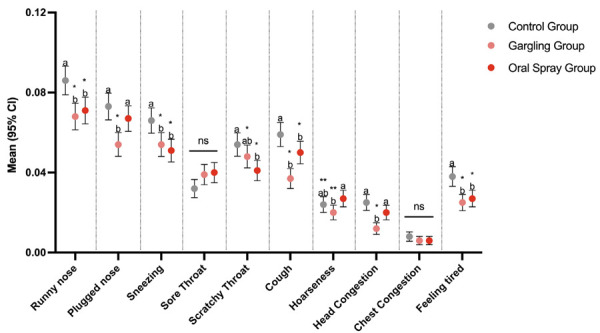
Mean values with 95% confidence interval of the severity of each cold symptom between groups. Ten different items were analyzed, and subjects were asked to indicate their level of agreement using a 7-point Likert scale over a 90-day period. Likert scale was converted to a binary score (0 = no symptom presented; 1 = symptom presented).

In addition to the overall status, individuals who presented symptoms filled out the Severity of Cold Symptoms questionnaire. Figure 3 demonstrates the presence of symptoms. The symptoms of a runny nose, sneezing, cough and feeling tired were demonstrated to be worse in the control group when compared with both test groups. Furthermore, with respect to a plugged nose, scratchy throat, hoarseness and head congestion, the control group presented worse scores when compared with either of the test groups. Sore throat and chest congestion did not differ among groups.

Different small letters indicate a statically significant difference between groups (*p ≤ 0.000; **p ≤ 0.05). Connected ns segments represent no statistically significant difference between groups. CI: confidence interval; ns: non-significant.

**Figure 4:**
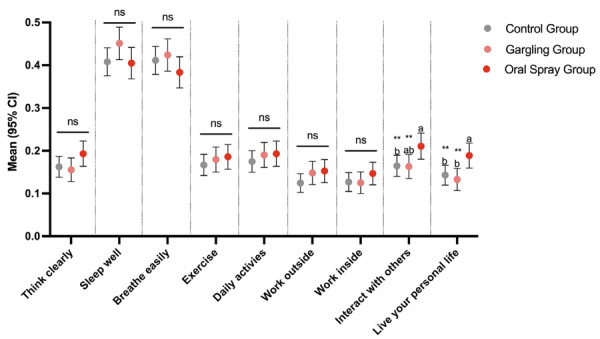
Mean values with 95% confidence interval of the cold ability to interfere in different daily activities. Nine different items were analyzed, and subjects were asked to indicate their level of agreement using a 7-point Likert scale over a 90-day period. The Likert scale was converted to a binary score (0 = not impacted; 1 = impacted).

The impact of the presence of upper respiratory symptoms in the previous 24 hours, was also evaluated and data are presented in Figure 4. In the majority of domains, no statistically significant differences were observed among groups (think clearly; sleep well; breathe easily; exercise; daily activities; work outside and work inside). Statistically significant differences were observed in the domains; interact with others and live your personal life, with higher means in the group that used spray.

Different small letters indicate a statically significant difference between groups (**p ≤ 0.05). Connected ns segments represent no statistically significant difference between groups. CI: confidence interval; n: non-significant.

## Discussion

The present randomized controlled clinical trial compared 2 regimens of extended oral hygiene for the occurrence and severity of upper respiratory symptoms in comparison with a negative control group. The novelty under study was that an antimicrobial agent and a cleaning strategy for the posterior region mouth could influence the occurrence of upper respiratory symptoms.

It is well known that upper respiratory symptoms account for a considerable amount in health care costs and impair quality of life.^13,14^ One of the main causes of upper respiratory symptoms is the seasonal flu. Viral infections affect individuals of all ages. In the present study, adults up to 69 years of age were included. This range was chosen in order to avoid extremely old individuals. No difference in age among groups was observed.

In this sense, seeking possibilities of diminishing the occurrence, severity and impact of upper respiratory symptoms is of great interest. Especially when viral infections are concerned, hygiene measures have been demonstrated to be one of the efficient tools in prevention. For example, washing hands has been one of the core practices in preventing a series of diseases.^15-17^With that in mind, and taking into consideration previous studies, we posed a hypothesis that using an oral antiseptic could potentially be of help to prevent upper respiratory symptoms.

Cetylpyridinium chloride is a cationic antiseptic that is safe, readily available as well as inexpensive and has been studied in health care for decades. The antibacterial properties of CPC are well known and potential antiviral properties have been reported.^18,19^Taking into consideration this potential, in the present study, we compared two forms of extending oral hygiene to include the whole oral cavity (gargling or using an oral spray) for their impact on the occurrence of upper respiratory symptoms. Both gargle and spray solutions are commercially available and contain CPC. The study was not performed with CPC+Zn solution since the spray does not contain Zinc and, therefore the comparison of the form of delivering (gargling or spraying) would not be fair.

This study was conducted during the time of the year that has most impact on for the topic under study. It is extremely common for overloading of the health system to occur, and completely filling the emergency services. Moreover, it should be remembered that during the study period, the COVID-19 pandemic was on the go. Taking into consideration these situations, we included a 25% attrition rate for the present study. The sample size estimate suggested 60 individuals per group and 75 were included. The number of individuals analyzed in each group was above 65 in all groups, warranting the estimate established.

The present study used a validated instrument - WURSS-21 (Wisconsin Upper Respiratory Symptom Survey-21). The aim of this instrument is to verify the daily occurrence of daily upper respiratory symptoms.^12^ The instrument was translated into Brazilian Portuguese, back translated to English and evaluated in terms of comprehension in a focus group comprising individuals similar to those included in the study. Compliance of the participants was checked weekly by requesting patients to provide images of the daily questionnaires they answered. Participants were also evaluated in terms of oral mucosal lesions, including staining of the mucosa, and no intercurrence was observed,. Therefore, it could be inferred that gargling and/or using an oral spray with CPC was not associated with extrinsic staining.

The results of the study demonstrated lower occurrence of respiratory symptoms with the use of CPC both by means of gargling or spraying. This was considered a regimen, since participants also performed their regular oral hygiene care. The possible mechanism by which this result occurred is that personal hygiene is associated with lower levels of different infections. Adding an antiseptic to regular oral care, has the potential of increasing the quality of oral hygiene. During the COVID-19 pandemic, 2 studies conducted at our University demonstrated that dental biofilm of individuals that were symptomatic for COVID-19 harbored SARS-COV-2, therefore also being a possible reservoir for re-infections. Thus, measures that also aim to combat oral biofilms have the possibility of preventing the occurrence and spread of viral infections.^20,21^

When considering the severity of symptoms, the results of the present study also demonstrated a promising effect when oral hygiene was extended to include the whole oral cavity since statistically significant effects were observed in comparison with the control group for several of the symptoms evaluated. When considering the impact of the upper respiratory symptoms , the present study was not able to demonstrate a tangible effect, which may also be a limitation of the instrument used. An additional avenue that merits further investigation is a possible correlation of the degree of bioburden and severity of symptoms.

Taking into consideration that gargling or using an oral spray decreased the occurrence and severity of upper respiratory symptoms, it could be postulated that the additional cleaning of the whole oral cavity (cheeks, mucosa, tongue, etc.) was the mechanism by which this effect was achieved. No significant difference could be detected between gargling and spraying. Taking into consideration that in an evidence-based healthcare practice, one of the pillars is the patient’s preference. This choice may be discussed among practitioners and patients. The decrease in respiratory infections has individual and collective benefits. Therefore, the benefits demonstrated herein transcend individual users.

The present study has strengths and limitations that need to be highlighted. Among the advantages, it should be emphasized that the study was conducted during the adequate season for studying respiratory diseases. Moreover, compliance was checked. In addition, the instrument was capable of evaluating upper respiratory symptoms and, therefore, reflected reality. Among the limitations, for obtainment of data, we relied on self-reports, which is always subjected to some level of criticism, in spite of the instrument having been ,validated and its capacity in detecting the information was reliable. However, memory bias could be present. It should be mentioned that the validation was not performed in Brazil. However, in a pilot study, we tested it for understanding. Furthermore, among possible limitations, the lack of microbiological sampling of the individuals included in the study. This would have been impossible on a daily basis during an extended period of study. In addition, diet and physical activity habits were not recorded and could interfere with the results. Whereas the fact of random allocation of participants would prevent any group from being identified. The present study used commercially available products and participating individuals could not be blinded to their allocation, which can be considered a potential source of bias. Additional studies in different parts of the world are warranted to confirm this potential benefit of cleaning the posterior region of the mouth and its impact on the occurrence of upper respiratory symptoms.

## Conclusion

In conclusion, considering the characteristics and limitations of the study of adding an additional cleansing step to enable the whole oral cavity to be reached with either gargling mouthwash or oral spray, has the potential to help to reduce the incidence of upper respiratory symptoms.

## Data Availability

The contents underlying the research text are contained in the manuscript.
